# Supplementary material for: Studies of the Formation of Inclusion Complexes Derivatives of Cinnamon Acid with α-Cyclodextrin in a Wide Range of Temperatures Using Conductometric Methods
Source: Molecules. 2022 Jul 10;27(14):4420. doi: 10.3390/molecules27144420 (PMC9318531; doi:10.3390/molecules27144420)
Supplement: Supplementary file 1 [file molecules-27-04420-s001.zip › molecules-1804469-supplementary.pdf]

Studies of the Formation of Inclusion Complexes Derivatives of Cinnamon Acid with α-Cyclodextrin in a Wide Range of Temperatures Using Conductometric Methods

Zdzisław Kinart [1,*] and Renato Tomaš [2]

[1] Department of Physical Chemistry, Faculty of Chemistry, University of Lodz, Pomorska 163/165, 90-236 Lodz, Poland
[2] Department of Physical Chemistry, University of Split, Ruđera Boškovića 35, HR-21000 Split, Croatia; renato.tomas@ktf-split.hr
* Correspondence: zdzislaw.kinart@chemia.uni.lodz.pl

Table S1. Densities, viscosities and relative permittivities of pure water at temperatures T = (288.15 to 318.15) K.

T/K	ρ_0 /kg·m^{-3} [a]	$10^3 \cdot \eta$ /Pa·s [a]	ε_r [b]
288.15	999.100	1.138	82.07
293.15	998.205	1.002	80.21
298.15	997.047	0.890	78.40
303.15	995.651	0.797	76.62
308.15	994.038	0.719	74.89
313.15	992.224	0.653	73.19
318.15	990.223	0.596	71.53

[a] values for water recommended by the International Association for the Properties of Water and Steam (IAPWS) calculated on the online property calculator (https://web1.hszg.de/thermo_fpc/). [b] values for water calculated according to the IAPWS recommendations (http://www.iapws.org/rel-guide/dielec.pdf).

Table S2. The value of molar concentration of salt C_{salt} [mol/dm^3], concentration of ligand C_{lig} [mol/dm^3], molar conductivity Λ [S·cm^2·mol^{-1}] for α-cyclodextrin with cinnamic acid sodium salt at a given molar fraction of water (x_w) in water at T = 288.15K at pressure p = 0.1 MPa.[a]

Sodium Salt of Cinnamic Acid			
Nr	c_{salt} [mol/dm^3]	c_{lig} [mol/dm^3]	Λ_m [S·cm^2·mol^{-1}]
1.	0.00269	0.00000	73.2734
2.	0.00263	0.00030	73.1178
3.	0.00257	0.00060	72.8742
4.	0.00251	0.00091	72.6399
5.	0.00244	0.00121	72.4521
6.	0.00239	0.00149	72.2658
7.	0.00232	0.00181	71.9855
8.	0.00226	0.00208	71.7513
9.	0.00221	0.00235	71.5558
10.	0.00214	0.00270	71.2835
11.	0.00208	0.00299	71.1018
12.	0.00202	0.00329	70.8764
13.	0.00195	0.00359	70.6802
14.	0.00189	0.00388	70.4438
15.	0.00183	0.00416	70.2263
16.	0.00177	0.00448	70.0045
17.	0.00171	0.00477	69.8121
18.	0.00164	0.00506	69.6401

19.	0.00159	0.00534	69.4027
20.	0.00153	0.00564	69.2034
21.	0.00147	0.00591	68.9945
22.	0.00141	0.00620	68.8742
23.	0.00134	0.00654	68.7909

[a] Standard uncertainties are $u(T)$ = 0.01 K, $u(p)$ = 0.05 MPa, $u(c) = 10^{-4}\cdot c$, and the combined expanded uncertainty is $U_c(\Lambda) = 0.0005\cdot\Lambda$ (level of confidence = 0.95).

Table S3. The value of molar concentration of salt C_{salt} [mol/dm³], concentration of ligand C_{lig} [mol/dm³],, molar conductivity Λ [S·cm²·mol⁻¹] for α-cyclodextrin with cinnamic acid sodium salt at a given molar fraction of water (x_w) in water at T = 293.15K.

**Sodium Salt of Cinnamic Acid**			
**Nr**	**c_{salt} [mol/dm³]**	**c_{lig} [mol/dm³]**	**Λ_m [S·cm²·mol⁻¹]**
1.	0.00288	0.00000	87.2346
2.	0.00281	0.00030	86.9317
3.	0.00275	0.00059	86.7459
4.	0.00268	0.00089	86.5075
5.	0.00261	0.00120	86.1627
6.	0.00255	0.00149	85.7303
7.	0.00248	0.00179	85.3416
8.	0.00242	0.00209	84.9778
9.	0.00235	0.00238	84.6581
10.	0.00229	0.00267	84.2786
11.	0.00222	0.00298	83.9136
12.	0.00215	0.00328	83.6255
13.	0.00209	0.00356	83.4177
14.	0.00202	0.00388	83.1784
15.	0.00195	0.00417	82.9643
16.	0.00189	0.00447	82.6906
17.	0.00182	0.00477	82.4736
18.	0.00176	0.00506	82.2882
19.	0.00169	0.00535	82.1220
20.	0.00162	0.00566	81.9316
21.	0.00156	0.00595	81.6714
22.	0.00149	0.00625	81.5324
23.	0.00143	0.00653	81.3958

Table S4. The value of molar concentration of salt C_{salt} [mol/dm³], concentration of ligand C_{lig} [mol/dm³],, molar conductivity Λ [S·cm²·mol⁻¹] for α-cyclodextrin with cinnamic acid sodium salt at a given molar fraction of water (x_w) in water at T = 298.15K.

**Sodium Salt of Cinnamic Acid**			
**Nr**	**c_{salt} [mol/dm³]**	**c_{lig} [mol/dm³]**	**Λ_m [S·cm²·mol⁻¹]**
1.	0.00291	0.00000	98.1387
2.	0.00284	0.00030	97.6825
3.	0.00277	0.00059	97.1099
4.	0.00271	0.00088	96.6581
5.	0.00265	0.00114	96.4077
6.	0.00257	0.00150	96.0937
7.	0.00250	0.00179	95.8108
8.	0.00243	0.00209	95.5416

9.	0.00237	0.00239	95.2654
10.	0.00230	0.00268	95.0197
11.	0.00223	0.00298	94.7489
12.	0.00217	0.00328	94.5165
13.	0.00210	0.00358	94.2562
14.	0.00203	0.00387	94.0001
15.	0.00197	0.00416	93.7400
16.	0.00190	0.00447	93.4926
17.	0.00184	0.00475	93.2690
18.	0.00178	0.00501	93.0345
19.	0.00171	0.00532	92.8006
20.	0.00164	0.00561	92.4950
21.	0.00157	0.00594	92.2153
22.	0.00151	0.00619	92.0167
23.	0.00145	0.00644	91.7749

Table S5. The value of molar concentration of salt C_{salt} [mol/dm^3], concentration of ligand C_{lig} [mol/dm^3],, molar conductivity Λ [S·cm^2·mol^{-1}] for α-cyclodextrin with cinnamic acid sodium salt at a given molar fraction of water (x_w) in water at T = 303.15K.

Sodium Salt of Cinnamic Acid			
Nr	c_{salt} [mol/dm^3]	c_{lig} [mol/dm^3]	Λ_m [S·cm^2·mol^{-1}]
1.	0.00292	0.00000	108.1296
2.	0.00285	0.00029	107.7095
3.	0.00278	0.00060	107.4119
4.	0.00271	0.00092	107.0464
5.	0.00265	0.00119	106.7432
6.	0.00259	0.00146	106.3876
7.	0.00251	0.00178	105.8817
8.	0.00245	0.00208	105.4283
9.	0.00238	0.00239	104.9864
10.	0.00231	0.00267	104.5927
11.	0.00225	0.00295	104.2338
12.	0.00217	0.00330	103.8056
13.	0.00211	0.00357	103.5472
14.	0.00205	0.00385	103.1529
15.	0.00198	0.00416	102.8244
16.	0.00191	0.00445	102.5493
17.	0.00185	0.00473	102.1688
18.	0.00178	0.00505	101.7869
19.	0.00171	0.00536	101.3329
20.	0.00165	0.00563	100.9962
21.	0.00158	0.00593	100.5672
22.	0.00152	0.00620	100.1848
23.	0.00145	0.00652	99.6309

Table S6. The value of molar concentration of salt C_{salt} [mol/dm^3], concentration of ligand C_{lig} [mol/dm^3],, molar conductivity Λ [S·cm^2·mol^{-1}] for α-cyclodextrin with cinnamic acid sodium salt at a given molar fraction of water (x_w) in water at T = 308.15K.

Sodium Salt of Cinnamic Acid			
**Nr**	**c_{salt} [mol/dm^3]**	**c_{lig} [mol/dm^3]**	**Λ_m [S·cm^2·mol^{-1}]**
1.	0.00287	0.00000	119.2278
2.	0.00281	0.00030	118.2364
3.	0.00276	0.00058	117.2351
4.	0.00270	0.00088	116.1811
5.	0.00264	0.00121	115.0497
6.	0.00259	0.00148	114.1814
7.	0.00253	0.00178	113.2525
8.	0.00248	0.00207	112.6112
9.	0.00242	0.00237	111.9282
10.	0.00236	0.00266	111.284
11.	0.00231	0.00294	110.7565
12.	0.00225	0.00324	110.2212
13.	0.00220	0.00354	109.7342
14.	0.00214	0.00385	109.3303
15.	0.00208	0.00416	108.9881
16.	0.00202	0.00446	108.749
17.	0.00197	0.00475	108.4971
18.	0.00191	0.00504	108.3266
19.	0.00185	0.00534	108.098
20.	0.00180	0.00563	107.9266
21.	0.00174	0.00592	107.8125
22.	0.00168	0.00623	107.6533
23.	0.00163	0.00651	107.5427

Table S7. The value of molar concentration of salt C_{salt} [mol/dm^3], concentration of ligand C_{lig} [mol/dm^3],, molar conductivity Λ [S·cm^2·mol^{-1}] for α-cyclodextrin with caffeic acid sodium salt at a given molar fraction of water (x_w) in water at T = 288.15K.

Sodium Salt of Caffeic Acid			
**Nr**	**c_{salt} [mol/dm^3]**	**c_{lig} [mol/dm^3]**	**Λ_m [S·cm^2·mol^{-1}]**
1.	0.00300	0.00000	50.6381
2.	0.00293	0.00029	50.5251
3.	0.00286	0.00062	50.3889
4.	0.00279	0.00089	50.3049
5.	0.00273	0.00119	50.1812
6.	0.00265	0.00151	50.0858
7.	0.00259	0.00179	49.9763
8.	0.00252	0.00210	49.8878
9.	0.00244	0.00238	49.7987
10.	0.00238	0.00270	49.6881
11.	0.00232	0.00298	49.5579
12.	0.00225	0.00328	49.4809
13.	0.00218	0.00359	49.3567
14.	0.00212	0.00388	49.2799
15.	0.00205	0.00418	49.2217
16.	0.00198	0.00446	49.1618

17.	0.00191	0.00478	49.1231
18.	0.00184	0.00508	49.0872
19.	0.00177	0.00538	49.0595
20.	0.00171	0.00565	49.0386
21.	0.00164	0.00597	49.0178
22.	0.00157	0.00626	48.9925
23.	0.00151	0.00656	48.9811

Table S8. The value of molar concentration of salt C_{salt} [mol/dm^3], concentration of ligand C_{lig} [mol/dm^3],, molar conductivity Λ [S·cm^2·mol^{-1}] for α-cyclodextrin with caffeic acid sodium salt at a given molar fraction of water (x_w) in water at T = 293.15K.

Sodium Salt of Caffeic Acid			
**Nr**	**c_{salt} [mol/dm^3]**	**c_{lig} [mol/dm^3]**	**Λ_m [S·cm^2·mol^{-1}]**
1.	0.00302	0.00000	57.6381
2.	0.00295	0.00030	57.5251
3.	0.00288	0.00060	57.3889
4.	0.00281	0.00091	57.3049
5.	0.00275	0.00120	57.1812
6.	0.00268	0.00150	57.0858
7.	0.00261	0.00178	56.9763
8.	0.00253	0.00212	56.8878
9.	0.00247	0.00240	56.7987
10.	0.00240	0.00270	56.6881
11.	0.00233	0.00299	56.5579
12.	0.00227	0.00328	56.4809
13.	0.00219	0.00359	56.3567
14.	0.00213	0.00387	56.2799
15.	0.00206	0.00417	56.2217
16.	0.00199	0.00447	56.1618
17.	0.00192	0.00478	56.1231
18.	0.00185	0.00507	56.0872
19.	0.00179	0.00537	56.0595
20.	0.00172	0.00566	56.0386
21.	0.00165	0.00594	56.0178
22.	0.00158	0.00625	55.9925
23.	0.00152	0.00653	55.9811

Table S9. The value of molar concentration of salt C_{salt} [mol/dm^3], concentration of ligand C_{lig} [mol/dm^3],, molar conductivity Λ [S·cm^2·mol^{-1}] for α-cyclodextrin with caffeic acid sodium salt at a given molar fraction of water (x_w) in water at T = 298.15K.

Sodium Salt of Caffeic Acid			
**Nr**	**c_{salt} [mol/dm^3]**	**c_{lig} [mol/dm^3]**	**Λ_m [S·cm^2·mol^{-1}]**
1.	0.00307	0.00000	65.0203
2.	0.00300	0.00030	64.9480
3.	0.00293	0.00061	64.8573
4.	0.00286	0.00090	64.7965
5.	0.00279	0.00120	64.7098
6.	0.00272	0.00148	64.6127
7.	0.00265	0.00181	64.5253
8.	0.00258	0.00209	64.4696

9.	0.00251	0.00239	64.3758
10.	0.00245	0.00269	64.3101
11.	0.00238	0.00298	64.2280
12.	0.00231	0.00329	64.1500
13.	0.00224	0.00358	64.0785
14.	0.00217	0.00388	64.0065
15.	0.00210	0.00417	63.9536
16.	0.00203	0.00448	63.8866
17.	0.00196	0.00477	63.8303
18.	0.00189	0.00507	63.7974
19.	0.00183	0.00535	63.7501
20.	0.00175	0.00566	63.7050
21.	0.00168	0.00596	63.6602
22.	0.00162	0.00624	63.6281
23.	0.00156	0.00651	63.6284

Table S10. The value of molar concentration of salt C_{salt} [mol/dm^3], concentration of ligand C_{lig} [mol/dm^3],, molar conductivity Λ [S·cm^2·mol^{-1}] for α-cyclodextrin with caffeic acid sodium salt at a given molar fraction of water (x_w) in water at T = 303.15K.

**Sodium Salt of Caffeic Acid**			
**Nr**	**c_{salt} [mol/dm^3]**	**c_{lig} [mol/dm^3]**	**Λ_m [S·cm^2·mol^{-1}]**
1.	0.00301	0.00000	72.2700
2.	0.00294	0.00030	72.2354
3.	0.00287	0.00060	72.1743
4.	0.00280	0.00092	72.1064
5.	0.00273	0.00120	72.0429
6.	0.00266	0.00150	71.9858
7.	0.00260	0.00179	71.9003
8.	0.00253	0.00210	71.8150
9.	0.00246	0.00238	71.7606
10.	0.00239	0.00270	71.6744
11.	0.00233	0.00298	71.5638
12.	0.00225	0.00329	71.4875
13.	0.00219	0.00357	71.4129
14.	0.00212	0.00388	71.3406
15.	0.00205	0.00417	71.2439
16.	0.00199	0.00447	71.1817
17.	0.001920	0.00476	71.1020
18.	0.00185	0.00505	71.0308
19.	0.00178	0.00535	70.9532
20.	0.00171	0.00568	70.9071
21.	0.00165	0.00592	70.8501
22.	0.00158	0.006250	70.7738
23.	0.00151	0.00653	70.7342

Table S11. The value of molar concentration of salt C_{salt} [mol/dm^3], concentration of ligand C_{lig} [mol/dm^3],, molar conductivity Λ [S·cm^2·mol^{-1}] for α-cyclodextrin with caffeic acid sodium salt at a given molar fraction of water (x_w) in water at T = 308.15K.

Sodium Salt of Caffeic Acid			
Nr	c_{salt} [mol/dm^3]	c_{lig} [mol/dm^3]	Λ_m [S·cm^2·mol^{-1}]
1.	0.00286	0.00000	80.0335
2.	0.00281	0.00030	78.7602
3.	0.00275	0.00059	77.4504
4.	0.00269	0.00089	76.1828
5.	0.00264	0.00117	75.0257
6.	0.00258	0.00148	73.9011
7.	0.00253	0.00177	72.8803
8.	0.00247	0.00207	71.9121
9.	0.00241	0.00236	70.9211
10.	0.00236	0.00266	70.0745
11.	0.00230	0.00296	69.4215
12.	0.00224	0.00326	68.6922
13.	0.00219	0.00355	68.1558
14.	0.00213	0.00384	67.6654
15.	0.00208	0.00415	67.2330
16.	0.00202	0.00444	66.9645
17.	0.00196	0.00473	66.6679
18.	0.00191	0.00502	66.4792
19.	0.00186	0.00530	66.1728
20.	0.00180	0.00561	65.9744
21.	0.00174	0.00590	65.7143
22.	0.00169	0.00619	65.5805
23.	0.00163	0.00649	65.4244

Table S12. The value of molar concentration of salt C_{salt} [mol/dm^3], concentration of ligand C_{lig} [mol/dm^3], molar conductivity Λ [S·cm^2·mol^{-1}] for α-cyclodextrin with coumaric acid sodium salt at a given molar fraction of water (x_w) in water at T = 288.15K.

Sodium Salt of Coumaric Acid			
Nr	c_{salt} [mol/dm^3]	c_{lig} [mol/dm^3]	Λ_m [S·cm^2·mol^{-1}]
1.	0.00291	0.00000	58.1464
2.	0.00284	0.00030	57.9332
3.	0.00277	0.00060	57.7233
4.	0.00271	0.00091	57.5203
5.	0.00264	0.00119	57.2468
6.	0.00258	0.00149	56.9505
7.	0.00251	0.00179	56.7181
8.	0.00244	0.00212	56.4919
9.	0.00238	0.00239	56.2914
10.	0.00231	0.00270	56.1217
11.	0.00225	0.00297	56.0039
12.	0.00218	0.00329	55.8160
13.	0.00212	0.00357	55.7434
14.	0.00205	0.00388	55.6168
15.	0.00199	0.00417	55.4599
16.	0.00192	0.00447	55.3430

17.	0.00185	0.00477	55.2500
18.	0.00179	0.00507	55.2037
19.	0.00172	0.00537	55.1576
20.	0.00165	0.00569	55.0964
21.	0.00159	0.00595	55.0929
22.	0.00153	0.00625	55.1204
23.	0.00146	0.00655	55.1342

Table S13. The value of molar concentration of salt C_{salt} [mol/dm^3], concentration of ligand C_{lig} [mol/dm^3], molar conductivity Λ [S·cm^2·mol^{-1}] for α-cyclodextrin with coumaric acid sodium salt at a given molar fraction of water (x_w) in water at T = 293.15K.

Sodium Salt of Coumaric Acid			
Nr	c_{salt} [mol/dm^3]	c_{lig} [mol/dm^3]	Λ_m [S·cm^2·mol^{-1}]
1.	0.00290	0.00000	65.5592
2.	0.00283	0.00030	65.4264
3.	0.00276	0.00060	65.1598
4.	0.00270	0.00089	64.9703
5.	0.00263	0.00119	64.7497
6.	0.00256	0.00149	64.5080
7.	0.00250	0.00179	64.2783
8.	0.00243	0.00211	64.0618
9.	0.00237	0.00239	63.8294
10.	0.00230	0.00269	63.6454
11.	0.00223	0.00299	63.4610
12.	0.00217	0.00327	63.2929
13.	0.00210	0.00359	63.1001
14.	0.00204	0.00387	62.9515
15.	0.00197	0.00418	62.8009
16.	0.00190	0.00449	62.6120
17.	0.00184	0.00477	62.4731
18.	0.00178	0.00507	62.3228
19.	0.00171	0.00536	62.1972
20.	0.00165	0.00565	62.0239
21.	0.00158	0.00596	61.9179
22.	0.00152	0.00624	61.8520
23.	0.00145	0.00655	61.7362

Table S14. The value of molar concentration of salt C_{salt} [mol/dm^3], concentration of ligand C_{lig} [mol/dm^3], molar conductivity Λ [S·cm^2·mol^{-1}] for α-cyclodextrin with coumaric acid sodium salt at a given molar fraction of water (x_w) in water at T = 288.15K.

Sodium Salt of Coumaric Acid			
Nr	c_{salt} [mol/dm^3]	c_{lig} [mol/dm^3]	Λ_m [S·cm^2·mol^{-1}]
1.	0.00289	0.00000	73.8331
2.	0.00282	0.00031	73.6059
3.	0.00276	0.00059	73.3532
4.	0.00269	0.00091	73.0815
5.	0.00262	0.00121	72.7960
6.	0.00256	0.00149	72.5252
7.	0.00249	0.00180	72.3323
8.	0.00243	0.00208	72.0969

9.	0.00236	0.00241	71.8773
10.	0.00230	0.00267	71.7017
11.	0.00223	0.00300	71.5864
12.	0.00217	0.00328	71.4389
13.	0.00210	0.00357	71.3077
14.	0.00204	0.00386	71.1868
15.	0.00197	0.00417	71.0388
16.	0.00190	0.00448	70.9094
17.	0.00184	0.00477	70.8220
18.	0.00177	0.00506	70.7068
19.	0.00171	0.00536	70.6333
20.	0.00164	0.00565	70.5678
21.	0.00158	0.00594	70.5123
22.	0.00152	0.00622	70.5086
23.	0.00145	0.00655	70.4987

Table S15. The value of molar concentration of salt C_{salt} [mol/dm^3], concentration of ligand C_{lig} [mol/dm^3], molar conductivity Λ [S·cm^2·mol^{-1}]for α-cyclodextrin with coumaric acid sodium salt at a given molar fraction of water (x_w) in water at T = 303.15K.

**Sodium Salt of Coumaric Acid**			
**Nr**	**c_{salt} [mol/dm^3]**	**c_{lig} [mol/dm^3]**	**Λ_m [S·cm^2·mol^{-1}]**
1.	0.00286	0.00000	82.9025
2.	0.00280	0.00030	82.7329
3.	0.00273	0.00059	82.5551
4.	0.00266	0.00090	82.3417
5.	0.00260	0.00118	82.1579
6.	0.00253	0.00150	81.9304
7.	0.00247	0.00178	81.7001
8.	0.00241	0.00207	81.4729
9.	0.00235	0.00235	81.3165
10.	0.00227	0.00269	81.0895
11.	0.00221	0.00297	80.8302
12.	0.00215	0.00328	80.6582
13.	0.00208	0.00359	80.4229
14.	0.00202	0.00387	80.2708
15.	0.00195	0.00416	80.1315
16.	0.00189	0.00446	79.9890
17.	0.00182	0.00475	79.8177
18.	0.00176	0.00505	79.6976
19.	0.00169	0.00534	79.6003
20.	0.00163	0.00564	79.5004
21.	0.00157	0.00593	79.4233
22.	0.00150	0.00623	79.3035
23.	0.00144	0.00652	79.2098

Table S16. The value of molar concentration of salt C_{salt} [mol/dm³], concentration of ligand C_{lig} [mol/dm³], molar conductivity Λ [S·cm²·mol⁻¹] for α-cyclodextrin with coumaric acid sodium salt at a given molar fraction of water (x_w) in water at T = 308.15K.

Sodium Salt of Coumaric Acid			
**Nr**	**c_{salt} [mol/dm³]**	**c_{lig} [mol/dm³]**	**Λ_m [S·cm²·mol⁻¹]**
1.	0.00287	0.00000	91.4678
2.	0.00281	0.00030	90.4764
3.	0.00276	0.00058	89.4751
4.	0.00270	0.00088	88.4211
5.	0.00264	0.00121	87.2897
6.	0.00259	0.00148	86.4214
7.	0.00253	0.00178	85.4925
8.	0.00248	0.00207	84.8512
9.	0.00242	0.00237	84.1682
10.	0.00236	0.00266	83.5240
11.	0.00231	0.00294	82.9965
12.	0.00225	0.00324	82.4612
13.	0.00220	0.00354	81.9742
14.	0.00214	0.00385	81.5703
15.	0.00208	0.00416	81.2281
16.	0.00202	0.00446	80.9890
17.	0.00197	0.00475	80.7371
18.	0.00191	0.00504	80.5666
19.	0.00185	0.00534	80.3380
20.	0.00180	0.00563	80.1666
21.	0.00174	0.00592	80.0525
22.	0.00168	0.00623	79.8933
23.	0.00163	0.00651	79.7827

Table S17. The value of molar concentration of salt C_{salt} [mol/dm³], concentration of ligand C_{lig} [mol/dm³], molar conductivity Λ [S·cm²·mol⁻¹] for α-cyclodextrin with ferulic acid sodium salt at a given molar fraction of water (x_w) in water at T = 288.15K.

Sodium Salt of Ferulic Acid			
**Nr**	**c_{salt} [mol/dm³]**	**c_{lig} [mol/dm³]**	**Λ_m [S·cm²·mol⁻¹]**
1.	0.00309	0.00000	48.1272
2.	0.00302	0.00031	48.0063
3.	0.00295	0.00061	47.9410
4.	0.00288	0.00091	47.8536
5.	0.00281	0.00121	47.7667
6.	0.00274	0.00151	47.6867
7.	0.00267	0.00181	47.5717
8.	0.00260	0.00212	47.4873
9.	0.00253	0.00240	47.3953
10.	0.00247	0.00269	47.3207
11.	0.00240	0.00299	47.2326
12.	0.00232	0.00329	47.1449
13.	0.00226	0.00359	47.0968
14.	0.00219	0.00388	47.0503
15.	0.00212	0.00418	46.9952
16.	0.00205	0.00449	46.9294

17.	0.00198	0.00478	46.8883
18.	0.00191	0.00507	46.8433
19.	0.00184	0.00537	46.8156
20.	0.00177	0.00565	46.7838
21.	0.00170	0.00598	46.7457
22.	0.00163	0.00627	46.7256
23.	0.00157	0.00654	46.6842

Table S18. The value of molar concentration of salt C_{salt} [mol/dm^3], concentration of ligand C_{lig} [mol/dm^3], molar conductivity Λ [S·cm^2·mol^{-1}] for α-cyclodextrin with ferulic acid sodium salt at a given molar fraction of water (x_w) in water at T = 293.15K.

Sodium Salt of Ferulic Acid			
Nr	c_{salt} [mol/dm^3]	c_{lig} [mol/dm^3]	Λ_m [S·cm^2·mol^{-1}]
1.	0.00315	0.00000	54.6693
2.	0.00307	0.00031	54.5096
3.	0.00300	0.00062	54.4267
4.	0.00292	0.00093	54.3564
5.	0.00286	0.00121	54.2878
6.	0.00279	0.00149	54.2188
7.	0.00272	0.00180	54.1584
8.	0.00264	0.00210	54.0794
9.	0.00257	0.00240	54.0304
10.	0.00250	0.00269	53.9411
11.	0.00243	0.00299	53.8733
12.	0.00236	0.00330	53.7789
13.	0.00229	0.00358	53.7073
14.	0.00222	0.00388	53.6208
15.	0.00215	0.00418	53.5545
16.	0.00208	0.00448	53.4780
17.	0.00201	0.00476	53.4339
18.	0.00193	0.00507	53.3850
19.	0.00186	0.00536	53.3599
20.	0.00179	0.00566	53.3377
21.	0.00172	0.00596	53.3265
22.	0.00165	0.00624	53.3121
23.	0.00159	0.00651	53.3025

Table S19. The value of molar concentration of salt C_{salt} [mol/dm^3], concentration of ligand C_{lig} [mol/dm^3], molar conductivity Λ [S·cm^2·mol^{-1}] for α-cyclodextrin with ferulic acid sodium salt at a given molar fraction of water (x_w) in water at T = 298.15K.

Sodium Salt of Ferulic Acid			
Nr	c_{salt} [mol/dm^3]	c_{lig} [mol/dm^3]	Λ_m [S·cm^2·mol^{-1}]
1.	0.00311	0.00000	61.7905
2.	0.00305	0.00027	61.7204
3.	0.00297	0.00061	61.5883
4.	0.00290	0.00090	61.4823
5.	0.00283	0.00120	61.3822
6.	0.00276	0.00150	61.2979
7.	0.00269	0.00180	61.1908
8.	0.00262	0.00210	61.1202

9.	0.00255	0.00239	61.0575
10.	0.00248	0.00269	60.9855
11.	0.00241	0.00300	60.9225
12.	0.00234	0.00328	60.8625
13.	0.00227	0.00358	60.8006
14.	0.00220	0.00387	60.7442
15.	0.00213	0.00417	60.6702
16.	0.00206	0.00447	60.5894
17.	0.00199	0.00477	60.5316
18.	0.00192	0.00506	60.4555
19.	0.00185	0.00538	60.4059
20.	0.00178	0.00566	60.3825
21.	0.00171	0.00594	60.3462
22.	0.00165	0.00621	60.2849
23.	0.00158	0.00650	60.2664

Table S20. The value of molar concentration of salt C_{salt} [mol/dm^3], concentration of ligand C_{lig} [mol/dm^3], molar conductivity Λ [S·cm^2·mol^{-1}] for α-cyclodextrin with ferulic acid sodium salt at a given molar fraction of water (x_w) in water at T = 303.15K.

Sodium Salt of Ferulic Acid			
**Nr**	**c_{salt} [mol/dm^3]**	**c_{lig} [mol/dm^3]**	**Λ_m [S·cm^2·mol^{-1}]**
1.	0.00315	0.00000	68.2239
2.	0.00308	0.00031	67.3534
3.	0.00301	0.00060	66.5502
4.	0.00293	0.00092	65.7565
5.	0.00286	0.00121	65.0636
6.	0.00279	0.00149	64.3062
7.	0.00272	0.00179	63.6592
8.	0.00265	0.00209	62.9865
9.	0.00258	0.00239	62.4143
10.	0.00251	0.00269	61.9531
11.	0.00244	0.00299	61.4591
12.	0.00237	0.00328	61.0355
13.	0.00230	0.00358	60.6761
14.	0.00223	0.00388	60.3862
15.	0.00216	0.00417	60.0971
16.	0.00208	0.00447	59.8251
17.	0.00201	0.00476	59.6954
18.	0.00194	0.00506	59.4812
19.	0.00187	0.00535	59.3611
20.	0.00180	0.00564	59.3122
21.	0.00173	0.00594	59.2907
22.	0.00167	0.00622	59.2558
23.	0.00159	0.00652	59.2256

Table S21. The value of molar concentration of salt C_{salt} [mol/dm³], concentration of ligand C_{lig} [mol/dm³], molar conductivity Λ [S·cm²·mol⁻¹] for α-cyclodextrin with ferulic acid sodium salt at a given molar fraction of water (x_w) in water at T = 308.15K.

Sodium Salt of Ferulic Acid			
**Nr**	c_{salt} **[mol/dm³]**	c_{lig} **[mol/dm³]**	Λ_m **[S·cm²·mol⁻¹]**
1.	0.00293	0.00000	74.8039
2.	0.00287	0.00030	73.9334
3.	0.00281	0.00060	73.1302
4.	0.00275	0.00090	72.3365
5.	0.00270	0.00118	71.6436
6.	0.00264	0.00149	70.8862
7.	0.00258	0.00179	70.2392
8.	0.00252	0.00208	69.5665
9.	0.00246	0.00238	68.9943
10.	0.00241	0.00266	68.5331
11.	0.00235	0.00296	68.0391
12.	0.00229	0.00326	67.6155
13.	0.00223	0.00356	67.2561
14.	0.00217	0.00385	66.9662
15.	0.00211	0.00415	66.6771
16.	0.00205	0.00444	66.4051
17.	0.00199	0.00474	66.2754
18.	0.00194	0.00502	66.0612
19.	0.00188	0.00532	65.9411
20.	0.00182	0.00560	65.8922
21.	0.00176	0.00590	65.8707
22.	0.00171	0.00609	65.8358
23.	0.00165	0.00648	65.8056
